# Supplementary material for: The Effect of Combined Training and Racing High-Speed Exercise History on Musculoskeletal Injuries in Thoroughbred Racehorses: A Systematic Review and Meta-Analysis of the Current Literature
Source: Animals (Basel). 2020 Nov 11;10(11):2091. doi: 10.3390/ani10112091 (PMC7696103; doi:10.3390/ani10112091)
Supplement: Supplementary file 1 [file animals-10-02091-s004.zip › animals-965278-Supplementary File 1.pdf]

Supplementary File 1: Search Strategy for Systematic Review
The literature search strategy for the systematic review to assess the effect of high-speed exercise history on musculoskeletal injuries in Thoroughbred racehorses.

All searches were performed in April 2019.

PubMed
((((((((fatal*) OR fracture*) OR wastage) OR musculoskeletal injury) OR "Cumulative Trauma Disorders"[Mesh]) OR ((("Epidemiology"[Mesh] AND "epidemiology"[Subheading])) OR epidemiolog*)) OR epidemiolog*)) AND ((racehorse*) OR ((racing) AND (((horse*) OR equine) OR ("Horses"[Mesh] AND "Equidae"[Mesh]))))) AND training

Scopus
(fatal* OR fracture* OR wastage OR musculoskeletal AND injury OR epidemiolog*) AND (((racehorse) OR (horse* OR equine OR equidae)) AND (training) AND NOT human)

Web of Science
(((fatal* OR fracture* OR wastage OR musculoskeletal injury OR epidemiolog*) AND (racehorse* OR horse* OR equine)) AND training)

EMBASE
(((fatal* OR fracture* OR wastage OR musculoskeletal injury OR epidemiolog*) AND (racehorse* OR horse* OR equine)) AND training)
