# Supplementary material for: The Effect of Combined Training and Racing High-Speed Exercise History on Musculoskeletal Injuries in Thoroughbred Racehorses: A Systematic Review and Meta-Analysis of the Current Literature
Source: Animals (Basel). 2020 Nov 11;10(11):2091. doi: 10.3390/ani10112091 (PMC7696103; doi:10.3390/ani10112091)
Supplement: Supplementary file 1 [file animals-10-02091-s004.zip › animals-965278-Supplementary File 2.pdf]

Study number	Authors	Year	Country	Title	Study type	Population	Included	Reason for exclusion†
1	Anthenill, L. A. et al	2010	USA	Comparison of macrostructural and microstructural bone features in Thoroughbred racehorses with and without midbody fracture of the proximal sesamoid bone	case control	Californian post mortem program	no	4,5
2	Anthenill, L. A. et al	2007	USA	Risk factors for proximal sesamoid bone fractures associated with exercise history and horseshoe characteristics in Thoroughbred racehorses	case control	Racing Thoroughbred horses, age 2 to 5 years, that were necropsied	yes	
3	Bailey, C. J. et al	1999	Australia	Impact of injuries and disease on a cohort of two- and three-year-old thoroughbreds in training	cohort prospective	Horses catalogued at a major yearling sale	no	4,5
4	Bani Hassan, E. et al	2016	Australia	Role of subchondral bone remodelling in collapse of the articular surface of Thoroughbred racehorses with palmar osteochondral disease	cross-sectional	Racing Thoroughbred horses	no	4,5
5	Bani Hassan, E. et al	2017	Australia	Prevalence of subchondral bone pathological changes in the distal metacarpi/metatarsi of racing Thoroughbred horses	cross-sectional	Thoroughbred racehorses that died or were euthanased on Melbourne metropolitan race tracks or at the University of Melbourne Equine Centre	no	4,5
6	Beccati, F. et al	2014	Italy	Morphologic radiographic study of the proximal sesamoid bones of the forelimb in thoroughbred racehorses in training	cross-sectional	2-year-old Thoroughbred racehorses in commercial training	no	3,4,5
7	Bogers, S. H. et al	2016	NZ	Quantitative comparison of bone mineral density characteristics of the distal epiphysis of third metacarpal bones from Thoroughbred racehorses with or without condylar fracture	case control	Archived limbs from Thoroughbred racehorses with a condylar fracture and control limbs from Thoroughbred racehorses without a condylar fracture	no	3,4,5
8	Bogers, S. H. et al	2014	NZ	Impact of race training on volumetric bone mineral density and its spatial distribution in the distal epiphysis of the third metatarsal bone of 2-year-old horses	case control	2-year-old Thoroughbred fillies	no	3
9	Bolwell, C. et al	2017	NZ	Epidemiology of Musculoskeletal Injury during Racing on New Zealand Racetracks 2005-2011	cohort retrospective	Thoroughbred flat race starts	no	2,5
10	Bolwell, C. et al	2012	NZ	Risk factors for interruptions to training occurring before the first trial start of 2-year-old Thoroughbred racehorses	cohort prospective	2-year-old racehorses	yes	
11	Bolwell, C. et al	2017	NZ	Commercial equine production in New Zealand. 3. The racing and sport industries	cross-sectional	Thoroughbred and Standardbred industries NZ	no	1,3,4,5
12	Boyde, A. and Firth, E. C.	2005	NZ	Musculoskeletal responses of 2-year-old Thoroughbred horses to early training. 8. Quantitative back-scattered electron scanning electron microscopy and confocal fluorescence microscopy of the epiphysis of the third metacarpal bone	cohort	2-year-old Thoroughbred fillies	no	3,4,5
13	Boyde, A. et al	2011	HK	Cartilage damage involving extrusion of mineralisable matrix from the articular calcified cartilage and subchondral bone	cross-sectional	Thoroughbred racehorses that were in, or had been retired from, active race training at the Hong Kong Jockey Club	no	3,4,5
14	Brown, H. R. et al	2019	USA	A thoroughbred racehorse with a unicortical palmar lateral condylar fracture returned to training 14 days after surgery: A hypothesis on the role of a single bone screw on crack propagation	case report	2-year-old Thoroughbred racehorse	no	1,4,5
15	Carrier, T. K. et al	1998	USA	Association between long periods without high-speed workouts and risk of complete humeral or pelvic fracture in thoroughbred racehorses: 54 cases (1991-1994)	case crossover	Thoroughbred racehorses in california part of PM program	no	4,5
16	Clegg, P. D.	2011	UK	Musculoskeletal disease and injury, now and in the future. Part 1: fractures and fatalities	narrative review	Thoroughbred racehorses in UK	no	1,4,5
17	Clegg, P. D.	2012	UK	Musculoskeletal disease and injury, now and in the future. Part 2: Tendon and ligament injuries	narrative review	Thoroughbred racehorses in UK	no	1,4,5
18	Cogger, N. et al	2008	Australia	Incidence rate of musculoskeletal injuries and determinants of time to recovery in young Australian Thoroughbred racehorses	cohort prospective	Young Thoroughbred horses in Australia	no	4,5
19	Cogger, N. et al	2008	Australia	Profiling training preparation in young Australian Thoroughbred racehorses	cohort prospective	2- and 3-year old Thoroughbred racehorses	no	4,5
20	Cogger, N. et al	2006	Australia	Risk factors for musculoskeletal injuries in 2-year-old Thoroughbred racehorses	cohort prospective	2-year-old Thoroughbred racehorses	yes	
21	Cohen, N. D. et al	1997	USA	Racing-related factors and results of prerace physical inspection and their association with musculoskeletal injuries incurred in Thoroughbreds during races	case control	Thoroughbred racehorses	no	2
22	Cruz, A. M. et al	2007	Canada	Epidemiologic characteristics of catastrophic musculoskeletal injuries in Thoroughbred racehorses	cohort retrospective	Thoroughbred racehorses with CMIs	no	4,5
23	Dallap, B. L. et al	1999	USA	Results of screw fixation combined with cortical drilling for treatment of dorsal cortical stress fractures of the third metacarpal bone in 56 Thoroughbred racehorses	case series	Thoroughbred racehorses	no	1,4,5
24	Davidson, E. J. and Martin, B. B.	2004	USA	Stress fracture of the scapula in two horses	case series	Throuoghbred racehorses	no	1,4,5
25	Davis, A. M. et al	2017	HK	Improved radiological diagnosis of palmar osteochondral disease in the Thoroughbred racehorse	cohort prospective	Thoroughbreds that had been retired from active race training at the HKJC within 2 months	no	4,5
26	DeLay, J.	2017	Canada	Postmortem findings in Ontario racehorses, 2003–2015	cohort retrospective	PM cases through the Ontario Death registry	no	1,4,5
27	Dimock, A. N. et al	2013	USA	Humeral stress remodelling locations differ in Thoroughbred racehorses training and racing on dirt compared to synthetic racetrack surfaces	cohort retrospective	Horses that presented for bone phase nuclear scintigraphy	no	4,5
28	Dyson, P. K. et al	2008	UK	Days lost from training by two- and three-year-old Thoroughbred horses: A survey of seven UK training yards	cohort prospective	Throughbred racehorses in training	no	4,5
29	Engiles, J. B. et al	2017	USA	A diagnostic pathologist’s guide to carpal disease in racehorses	narrative review	Postmortem examination	no	1,4,5
30	Estberg, L. et al	1998	USA	A case-crossover study of intensive racing and training schedules and risk of catastrophic musculoskeletal injury and lay-up in California thoroughbred racehorses	case crossover	All racehorses which died or were euthanized on a California racetrack for a musculoskeletal injury incurred during racing or training.	no	4,5
31	Estberg, L. et al	1995	USA	Cumulative racing-speed exercise distance cluster as a risk factor for fatal musculoskeletal injury in Thoroughbred racehorses in California	case control	All racehorses which died or were euthanized on a California racetrack for a musculoskeletal injury incurred during racing or training.	no	4,5
32	Estberg, L. et al	1996	USA	High-speed exercise history and catastrophic racing fracture in Thoroughbreds	case control	Thoroughbreds racing in California	no	2

33	Estberg, L. et al	1996	USA	Fatal musculoskeletal injuries incurred during racing and training in thoroughbreds	case control	Thoroughbreds racing in California	no	4,5
34	Firth, E. C. et al	2009	NZ	Changes in mineralised tissue at the site of origin of condylar fracture are present before athletic training in Thoroughbred horses	non-randomised trial	Thoroughbred foals	no	1,3,4,5
35	Firth, E. C. and Rogers, C. W.	2005	NZ	Musculoskeletal responses of 2-year-old thoroughbred horses to early training. Conclusions	non-randomised trial	Throughbred fillies	no	1,3
36	Firth, E. C. and Rogers, C. W.	2005	NZ	Musculoskeletal responses of 2-year-old Thoroughbred horses to early training. 7. Bone and articular cartilage response in the carpus	non-randomised trial	Throughbred fillies	no	1,3
37	Firth, E. C. et al	2004	NZ	Musculoskeletal responses of 2-year-old Thoroughbred horses to early training. 4. Morphometric, microscopic and biomechanical properties of the digital tendons of the forelimb	non-randomised trial	Throughbred fillies	no	1,3
38	Firth, E. C. et al	2005	NZ	Musculoskeletal responses of 2-year-old Thoroughbred horses to early training. 6. Bone parameters in the third metacarpal and third metatarsal bones	non-randomised trial	Throughbred fillies	no	1,3
39	Firth, E. C. et al	2004	NZ	Musculoskeletal responses of 2-year-old Thoroughbred horses to early training. 1. Study design, and clinical, nutritional, radiological and histological observations	non-randomised trial	Throughbred fillies	no	1,3
40	Firth, E. C. et al	2007	NZ	Changes in diaphyseal and epiphyseal bone parameters in thoroughbred horses after withdrawal from training	non-randomised trial	Throughbred fillies	no	3
41	Frisbie, D. D. et al	2010	USA	Serum biomarker levels for musculoskeletal disease in two- and three-year-old racing Thoroughbred horses: A prospective study of 130 horses	cohort prospective	Two- and 3-year-old racehorses in California	no	4,5
42	Fugazzola, M. C. et al	2015	Italy	Correlation Between the Conformation of the Distal Forelimb and Superficial Digital Flexor Tendon Lesions in Flat Racing Thoroughbreds	cross-sectional	4-yearold Thoroughbred racehorses	no	4,5
43	Galuppo, L. D. et al	2006	USA	A clinical evaluation of a headless, titanium, variable-pitched, tapered, compression screw for repair of nondisplaced lateral condylar fractures in thoroughbred racehorses	case series	Thoroughbred racehorses	no	1,4,5
44	Georgopoulos, S et al	2017	USA	Risk factors for equine fractures in Thoroughbred flat racing in North America	cohort retrospective	Racecourses reporting injuries to the Equine Injury Database	no	2
45	Gibbons, A.	2014	USA	Racing for disaster?	editorial	International racehorses	no	1,3,4,5
46	Graham, R. J. et al	2015	HK	Retrospective study on the effect of wither fracture on racing performance in Thoroughbred racehorses in Hong Kong (2003 to 2013)	cohort retrospective	Racehorses in Hong Kong	no	1,4,5
47	Hanie, E. A. et al	1992	USA	Follow-up of 28 horses with third metacarpal unicortical stress fractures following treatment with osteostixis	case series	Thoroughbred racehorses	no	1,4,5
48	Hennessy, S. E. et al	2013	Australia	Effect of displaced versus non-displaced pelvic fractures on long-term racing performance in 31 Thoroughbred racehorses	case series	Thoroughbred racehorses	no	1,4,5
49	Hernandez, J. A. et al	2005	USA	Evaluation of horseshoe characteristics and high-speed exercise history as possible risk factors for catastrophic musculoskeletal injury in Thoroughbred racehorses	case control	Thoroughbred horses racing in Florida	no	2
50	Hesse, K. L. and Verheyen, K. L.	2010	UK	Associations between physiotherapy findings and subsequent diagnosis of pelvic or hindlimb fracture in racing Thoroughbreds	case control	Thoroughbred racehorses Newmarket	no	4,5
51	Hill, A. E. et al	2003	USA	Evaluation of a stochastic Markov-chain model for the development of forelimb injuries in Thoroughbred racehorses	report	Throughbred racehorses California	no	4,5
52	Hill, A. E. et al	2004	USA	Effects of injury to the suspensory apparatus, exercise, and horseshoe characteristics on the risk of lateral condylar fracture and suspensory apparatus failure in forelimbs of Thoroughbred racehorses	cross-sectional	Bilateral forelimb specimens distal to the antebrachiocarpal joint	yes	
53	Hill, A. E. et al	2001	USA	Risk factors for and outcomes of noncatastrophic suspensory apparatus injury in Thoroughbred racehorses	nested case control	Thoroughbred racehorse trainers who had horses entered in races at the Santa Anita and Hollywood Park racetracks California	no	5
54	Hill, W. T.	2003	USA	Survey of injuries in thoroughbreds at the New York Racing Association tracks	descriptive	New York racehorses	no	1,2,4,5
55	Hirsch, J. E. et al	2007	USA	Clinical evaluation of a titanium, headless variable-pitched tapered cannulated compression screw for repair of frontal plane slab fractures of the third carpal bone in thoroughbred racehorses	case series	Thoroughbred racehorses	no	1,4,5
56	Hitchens, P. L. et al	2018	USA	Relationship between historical lameness, medication usage, surgery, and exercise with catastrophic musculoskeletal injury in racehorses	case control	Thoroughbred racehorses in California	yes	
57	Hitchens, P. L. et al	2019	Australia	Meta-analysis of risk factors for racehorse catastrophic musculoskeletal injury in flat racing	meta-analysis	Thoroughbred racehorses	no	1,2
58	Holmes, J. M. et al	2014	Australia	Thoroughbred horses in race training have lower levels of subchondral bone remodelling in highly loaded regions of the distal metacarpus compared to horses resting from training	cross-sectional	Thoroughbred horses that died or were euthanased that underwent post-mortem examination at the University of Melbourne	no	3,4,5
59	Horseman, S. V. et al	2016	UK	Current Welfare Problems Facing Horses in Great Britain as Identified by Equine Stakeholders	cross-sectional	Interviewees were recruited across all four ofthese categories and a cross-section of disciplines including leisure riding, show jumping, dressage, eventing and racing	no	1,3,4,5
60	Jacklin, B. D. and Wright, I. M.	2012	UK	Frequency distributions of 174 fractures of the distal condyles of the third metacarpal and metatarsal bones in 167 Thoroughbred racehorses (1999-2009)	case series	Horses with fractures of the distal condyles of third metacarpal/metatarsal bones seen over the last 10 years at Newmarket Equine Hospital	no	1,4,5
61	Jackson, B. F. et al	2009	UK	Bone biomarkers and risk of fracture in two- and three-year-old Thoroughbreds	cohort prospective	2 and 3yo Thoroughbreds at 12 different training establishments in England	no	4,5
62	Jackson, B. F. et al	2003	UK	Relationship between bone markers, age at start of training, and risk of fracture and fatigue injury of the third metacarpal bone in two year old racehorses	cohort prospective	Thoroughbred racehoress in UK	no	1,4,5
63	Jackson, B. F. et al	2005	UK	Biochemical markers of bone metabolism and risk of dorsal metacarpal disease in 2-year-old Thoroughbreds	cohort prospective	Two-year-old Thoroughbreds in England	no	4,5
64	Janes, J. G. et al	2017	USA	Common lesions of the distal end of the third metacarpal/metatarsal bone in racehorse catastrophic breakdown injuries	narrative review	Research on catastrophic musculoskeletal injuries	no	1,4,5

65	Jeffcott, L. B. et al	1982	UK	An assessment of wastage in Thoroughbred racing from conception to 4 years of age	cross-sectional	Training stables in Newmarket	no	1,4,5
66	Johnson, B. J. et al	1994	USA	Causes of death in racehorses over a 2 year period	cohort retrospective	Racehorses that had a fatal injury or illness at a California racetrack	no	4,5
67	Kamm, J. L. et al	2011	USA	Size and geometry of apical sesamoid fracture fragments as a determinant of prognosis in Thoroughbred racehorses	case series	Weanlings, yearlings and racehorses that underwent surgery to remove apical PSB fractures	no	1,4,5
68	Kaneko, M. et al	1996	Japan	Morbid anatomy of soft tissue in fractured forelimbs of thoroughbred racehorses	case control	Thoroughbred racehorses	no	4,5
69	Kasashima, Y. et al	2004	Japan	Prevalence of superficial digital flexor tendonitis and suspensory desmitis in Japanese Thoroughbred flat racehorses in 1999	cohort retrospective	Thoroughbred racehorses	no	4,5
70	Kristoffersen, M. et al	2010	UK	Catastrophic biaxial proximal sesamoid bone fractures in UK Thoroughbred races (1999-2004): Horse characteristics and racing history	nested case control	Thoroughbreds racing at all 59 racecourses in the UK	no	2,4,5
71	Lindsay, W. A. et al	1982	USA	Management of slab fractures of the third tarsal bone in 5 horses	case series	Five cases of fracture of the third tarsal bone in racehorses are reported	no	1,4,5
72	Mackey, V. S. et al	1987	USA	Stress fractures of the humerus, radius and tibia in horses: Clinical Features and Radiographic and/or Scintigraphic Appearance	case series	Throughbred racehorses	no	1,4,5
73	MacKinnon, M. C. et al	2015	Canada and USA	Analysis of stress fractures associated with lameness in Thoroughbred flat racehorses training on different track surfaces undergoing nuclear scintigraphic examination	case series	Thoroughbred flat racehorses in active race training	no	1,4,5
74	Maeda, Y. et al	2016	Japan	Epidemiology of racing injuries in Thoroughbred racehorses with special reference to bone fractures: Japanese experience from the 1980s to 2000s	narrative review	Racing fractures that occurred on racetracks of the Japan Racing Association	no	1,4,5
75	Markel, M. D. and Richardson, D. W.	1985	USA	Noncomminuted fractures of the proximal phalanx in 69 horses	case series	Horses with noncomminuted fractures of the proximal phalanx, excluding proximal chip fractures	no	1,4,5
76	Marr, C. M.	2011	UK	The Horserace Betting Levy Board: 50 years of advances in equine veterinary science, education and practice	editorial	Horserace Betting Levy Board	no	1,3,4,5
77	Martig, S. et al	2014	Australia	Bone fatigue and its implications for injuries in racehorses	narrative review	Racehorses	no	1,3,4,5
78	Martig, S. et al	2018	Australia	Subchondral bone morphology in the metacarpus of racehorses in training changes with distance from the articular surface but not with age	cross-sectional	Thoroughbred racehorses from Victoria, Australia, that had died or were euthanised for reasons unrelated to this study	no	3,4,5
79	Martig, S. et al	2013	Australia	Compressive fatigue life of subchondral bone of the metacarpal condyle in thoroughbred racehorses	non-randomised trial	Thoroughbred racehorses in training that died or were euthanatized on racetracks in Victoria, Australia, during racing or training	no	3,4,5
80	Martin, G. S. et al	1988	USA	Effect of third carpal slab fracture and repair on racing performance in Thoroughbred horses: 31 cases (1977-1984)	case series	Thoroughbred horses	no	1,4,5
81	McGlinchey, L. et al	2017	HK	Description of the incidence, clinical presentation and outcome of proximal limb and pelvic fractures in Hong Kong racehorses during 2003–2014	cohort retrospective	Racehorses in Hong Kong	no	1,4,5
82	McIlwraith, C. W. et al	1987	USA	Arthroscopic surgery for the treatment of osteochondral chip fractures in the equine carpus	case series	Horses undergoing arthroscopic surgery for the removal of osteochondral fragments from carpal joints	no	1,4,5
83	Meagher, D. M. et al	2013	USA	Prevalence of abnormal radiographic findings in 2-year-old Thoroughbreds at in-training sales and associations with racing performance	cohort retrospective	Thoroughbreds at training sales in USA	no	4,5
84	Mizobe, F. et al	2019	Japan	Standing magnetic resonance imaging of distal phalanx fractures in 6 cases of Thoroughbred racehorse	case series	Thoroughbred racehorses	no	1,4,5
85	Mizobe, F. et al	2017	Japan	Signal changes in standing magnetic resonance imaging of osseous injury at the origin of the suspensory ligament in four Thoroughbred racehorses under tiludronic acid treatment	case series	Thoroughbred racehorses	no	1,4,5
86	Mizobe, F. et al	2019	Japan	Bone marrow oedema-type signal in the proximal phalanx of Thoroughbred racehorses	case series	Thoroughbred racehorses	no	1,4,5
87	Mizuno, Y.	1996	Japan	Fractures of the carpus in racing thoroughbreds of the Japanracing association: Prevalence, location, and current modes of surgicaltherapy	cohort retrospective	Japanese racehorses	no	1,4,5
88	Moffat, P. A. et al	2008	NZ	The influence of exercise during growth on ultrasonographic parameters of the superficial digital flexor tendon of young Thoroughbred horses	non-randomised trial	Thoroughbred foals	no	3
89	Moiroud, C. H. et al	2019	France	Distribution of Pelvic Fractures in Racing and Non-racing Sport Horses: A Retrospective Study of 86 Cases Examined in a Referral Centre	case series	All horses diagnosed with a pelvic fracture	no	1,4,5
90	More, S. J.	1999	Australia	A longitudinal study of racing thoroughbreds: performance during the first years of racing	cohort prospective	Thoroughbred horses in south-eastern Queensland	no	2,3,4,5
91	Morrice-West, A. V. et al	2018	Australia	Track Surfaces Used for Ridden Workouts and Alternatives to Ridden Exercise for Thoroughbred Horses in Race Training	cross-sectional	Thoroughbred trainers in Victoria	no	1,3,4,5
92	Moyer, W. et al	1991	USA	Relative incidence of dorsal meatacarpal disease in young Thoroughbred racehorses training on two different surfaces	cohort prospective	Thoroughbred racehorses	no	5
93	Murray, R. C. et al	2007	UK	How does exercise intensity and type affect equine distal tarsal subchondral bone thickness?	cross-sectional	Cadaver tarsi	no	3,4,5
94	Noble, P. et al	2016	UK	Does subchondral bone of the equine proximal phalanx adapt to race training?	cross-sectional	First phalangeal bones post-mortem	no	3,4,5
95	Nunamaker, D. M. et al	1989	USA	Some geometric properties of the third metacarpal bone: A comparison between the Thoroughbred and Standardbred racehorse	cross-sectional	Second, third, and fourth metacarpal bones	no	1,3,4,5
96	Nunamaker, D. M. et al	1990	USA	Fatigue fractures in thoroughbred racehorses: Relationships with age, peak bone strain, and training	non-randomised trial	Thoroughbred racehorses	no	1,5
97	Oikawa, M. and Kusunose, R.	2005	Japan	Fractures sustained by racehorses in Japan during flat racing with special reference to track condition and racing time	cohort prospective	Throughbred racehorses in Japan	no	2,5
98	Oki, H. et al	2008	Japan	Estimation of heritability for superficial digital flexor tendon injury by Gibbs sampling in the Thoroughbred racehorse	cohort retrospective	Japanese racehorses training	no	4,5
99	Olivier, A. et al	1997	South Africa	An epizoological study of wastage in thoroughbred racehorses in Gauteng, South Africa	cohort prospective	Throughbred racehorses	no	4,5
100	Parkin, T. D.	2007	UK	Epidemiology of training and racing injuries	narrative review	Thoroughbred injuries and fatalities	no	1,4,5

101	Parkin, T. D.	2008	UK	Epidemiology of racetrack injuries in racehorses	narrative review	Thoroughbred injuries and fatalities	no	1,2,4,5
102	Parkin, T. D. and Rossdale, P. D.	2006	UK	Epidemiology of equine performance wastage: importance of analysing facts and implementing their message in management	narrative review	Thoroughbred injuries and fatalities	no	1,4,5
103	Parkin, T. D. H. et al	2004	UK	Horse-level risk factors for fatal distal limb fracture in racing Thoroughbreds in the UK	case control	Thoroughbreds on UK racecourses	no	2
104	Parkin, T. D. H. et al	2005	UK	Risk factors for fatal lateral condylar fracture of the third metacarpus/metatarsus in UK racing	case control	Thoroughbreds on UK racecourses	no	2
105	Perkins, N. R. et al	2004	NZ	Effect of training location and time period on racehorse performance in New Zealand. 1. Descriptive analysis	cohort retrospective	Racehorses training and racing in New Zealand	no	3,4,5
106	Perkins, N. R. et al	2004	NZ	Effect of training location and time period on racehorse performance in New Zealand. 2. Multivariable analysis	cohort retrospective	Racehorses training and racing in New Zealand	no	3,4,5
107	Perkins, N. R. et al	2004	NZ	Profiling the New Zealand thoroughbred racing industry. 2. Conditions interfering with training and racing	cohort prospective	Racehorses training and racing in New Zealand	no	4,5
108	Perkins, N. R. et al	2004	NZ	Profiling the New Zealand thoroughbred racing industry. 1. Training, racing and general health patterns	cohort prospective	Racehorses training and racing in New Zealand	no	4,5
109	Perkins, N. R. et al	2005	NZ	Risk factors for musculoskeletal injuries of the lower limbs in Thoroughbred racehorses in New Zealand	cohort prospective	Racehorses training and racing in New Zealand	yes	
110	Perkins, N. R. et al	2005	NZ	Risk factors for injury to the superficial digital flexor tendon and suspensory apparatus in Thoroughbred racehorses in New Zealand	cohort prospective	Racehorses training and racing in New Zealand	no	5
111	Perkins, N. R. et al	2004	NZ	Musculoskeletal responses of 2-year-old Thoroughbred horses to early training. 3. In vivo ultrasonographic assessment of the cross-sectional area and echogenicity of the superficial digital flexor tendon	non-randomised trial	Thoroughbred fillies	no	3
112	Physick-Sheard, P. W. et al	2019	Canada	Ontario Racehorse Death Registry, 2003-2015: Descriptive analysis and rates of mortality	cohort retrospective	The Ontario Death Registry	no	1,4,5
113	Pilsworth, R. C. et al	1994	UK	Fracture of the wing of the ilium, adjacent to the sacroiliac joint, in thoroughbred racehorses	case series	Thoroughbred racehorses	no	1,4,5
114	Plevin, S. and McLellan, J.	2014	USA	Does periosteal scraping of the third metacarpal bone reduce the incidence of 'bucked shins' in young Thoroughbred racehorses?	cohort prospective	Yearling Thoroughbred racehorses	no	1,5
115	Plevin, S. et al	2019	USA	Ultrasound tissue characterisation of the superficial digital flexor tendons in juvenile Thoroughbred racehorses during early race training	cohort	Thirty-two TB yearling racehorses were recruited	no	1
116	Pool, R. R. and Meagher, D. M.	1990	USA	Pathologic findings and pathogenesis of racetrack injuries	narrative review	Racetrack injuries	no	1,2,4,5
117	Ramzan, P. H. L.	2009	UK	Transverse stress fracture of the distal diaphysis of the third metacarpus in six Thoroughbred racehorses	case series	Thoroughbred racehorses in flat race training	no	1,4,5
118	Ramzan, P. H. L. and Palmer, L.	2011	UK	Musculoskeletal injuries in Thoroughbred racehorses: A study of three large training yards in Newmarket, UK (2005-2007)	cohort prospective	Thoroughbred racehorses in UK	no	1,4,5
119	Ramzan, P. H. L. et al	2013	UK	Subclinical ultrasonographic abnormalities of the suspensory ligament branch of the athletic horse: A survey of 60 Thoroughbred racehorses	cross-sectional	Thoroughbred racehorses in UK	no	3,4,5
120	Reed, S. R. et al	2012	UK	Descriptive epidemiology of joint injuries in Thoroughbred racehorses in training	cohort prospective	Thoroughbred racehorses in UK	no	4,5
121	Reed, S. R. et al	2013	UK	Exercise affects joint injury risk in young Thoroughbreds in training	cohort prospective	Thoroughbred racehorses in UK	yes	
122	Riggs, C. M.	2002	Australia	Fractures - A preventable hazard of racing thoroughbreds?	narrative review	Throughbred racehorses	no	1,4,5
123	Riggs, C. M.	2019	HK	Computed tomography in equine orthopaedics – the next great leap?	editorial	equine orthopaedics	no	1,4,5
124	Riggs, C. M. and Boyde, A.	1999	UK	Effect of exercise on bone density in distal regions of the equine third metacarpal bone in 2-year-old thoroughbreds	non-randomised trial	Thoroughbred, unbroken fiilies	no	3
125	Riggs, C. M. et al	1999	UK	Structural variation of the distal condyles of the third metacarpal and third metatarsal bones in the horse	cross-sectional	Thoroughbred horses and an Arab foal subjected to euthanasia for a variety of clinical conditions	no	3,4,5
126	Riggs, C. M. et al	1999	UK	Pathology of the distal condyles of the third metacarpal and third metatarsal bones of the horse	cross-sectional	Distal limbs were collected from Thoroughbred horses destroyed for a variety of clinical conditions the majority of which had been in race training	no	3,4,5
127	Rijkenhuizen, A. B. et al	2012	Multicentre international	Management and outcome of fractures of the distal phalanx: A retrospective study of 285 horses with a long term outcome in 223 cases	case series	horses with distal phalangeal fractures	no	1,4,5
128	Robinson, R. A. et al	1988	USA	Epidemiological studies of musculoskeletal racing and training injuries in Thoroughbred horses, Minnesota, U.S.A	cohort prospective	Thoroughbred racehorses racing and training in Minnesota	no	1,4,5
129	Rogers, C. W. et al	2012	NZ	Early exercise in the horse	narrative review	Equine athletes	no	1,3,4,5
130	Rogers, C. W. and Firth, E. C.	2004	NZ	Musculoskeletal responses of 2-year-old Thoroughbred horses to early training. 2. Measurement error and effect of training stage on the relationship between objective and subjective criteria of training workload	non-randomised trial	2yo Throughbred fillies	no	3
131	Rogers, C. W. et al	2005	NZ	Musculoskeletal responses of 2-year-old thoroughbred horses to early training. 5. Kinematic effects	non-randomised trial	2yo Throughbred fillies	no	3
132	Rogers, C. W. et al	2008	NZ	Evaluation of a new strategy to modulate skeletal development in racehorses by imposing track-based exercise during growth: the effects on 2- and 3-year-old racing careers	non-randomised trial	Thoroughbred foals	no	3
133	Rosanowski, S. M. et al	2017	UK	Descriptive epidemiology of veterinary events in flat racing Thoroughbreds in Great Britain (2000 to 2013)	cohort retrospective	Thoroughbred racehorses participating in flat racing in the UK	no	1,2
134	Rosanowski, S. M. et al	2019	UK	Epidemiology of race-day distal limb fracture in flat racing Thoroughbreds in Great Britain (2000–2013)	cohort retrospective	Thoroughbred racehorses participating in flat racing in the UK	no	2
135	Rossdale, P. D. et al	1985	UK	Epidemiological study of wastage among racehorses 1982 and 1983	cohort prospective	Thoroughbred horses training in UK yards	no	1,4,5
136	Russell, T. M. and Maclean, A. A.	2006	Australia	Standing surgical repair of propagating metacarpal and metatarsal condylar fractures in racehorses	case series	Racehorses with a spiral/propagating condylar fracture	no	1,4,5
137	Rutherford, D. J. et al	2007	NZ	Outcome of lag-screw treatment of incomplete fractures of the frontal plane of the radial facet of the third carpal bone in horses	case series	Racehorses with incomplete C3 fractures	no	1,4,5
138	Sherman, K. M. et al	1995	USA	The effect of training on equine metacarpal bone breaking strength	non-randomised trial	Thoroughbreds, 24 to 48 months old in race training	no	3,4,5

139	Shimozawa, K. et al	2001	Japan	Survey of arthroscopic surgery for carpal chip fractures in thoroughbred racehorses in Japan	case series	Thoroughbred racehorses that underwent arthroscopic surgery for carpal chip fractures	no	1,4,5
140	Smith, L. C. R. et al	2009	UK	A lateral approach for screw repair in lag fashion of spiral third metacarpal and metatarsal medial condylar fractures in horses	case series	Thoroughbred racehorses with nondisplaced medial MC3/MT3 condylar fractures	no	1,4,5
141	Smith, L. C. R. et al	2018	UK	A longitudinal study of fractures in 1488 Thoroughbred racehorses receiving intrasynovial medication: 2006–2011	cohort retrospective	All Thoroughbred flat racehorses receiving intrasynovial medication under the first-opinion care of a single Newmarket, United Kingdom veterinary practice	no	4,5
142	Smith, M. R. and Wright, I. M.	2014	UK	Radiographic configuration and healing of 121 fractures of the proximal phalanx in 120 Thoroughbred racehorses (2007-2011)	case series	Thoroughbred racehorses from Newmarket Hospital with radiologically confirmed parasagittal fracture of the proximal phalanx	no	1,4,5
143	Smith, M. R. W. et al	2017	UK	Parasagittal fractures of the proximal phalanx in Thoroughbred racehorses in the UK: Outcome of repaired fractures in 113 cases (2007–2011)	case series	Thoroughbred racehorses admitted to Newmarket Equine Hospital for evaluation of a parasagittal fracture of the proximal phalanx during a 5 years period	no	1,4,5
144	Smith, R. K. et al	1999	UK	Should equine athletes commence training during skeletal development?: changes in tendon matrix associated with development, ageing, function and exercise	narrative review	Athletic horses	no	1
145	Stover, S. M.	2003	USA	The epidemiology of thoroughbred racehorse injuries	narrative review	Thoroughbred racehorses	no	1,4,5
146	Stover, S. M.	2017	USA	Nomenclature, classification, and documentation of catastrophic fractures and associated preexisting injuries in racehorses	narrative review	Racehorses	no	1,4,5
147	Stover, S. M. et al	1992	USA	An association between complete and incomplete stress fractures of the humerus in racehorses	case series	Horses training or racing at a California racetrack	no	1
148	Sun, T. C. et al	2019	HK	Noncatastrophic and catastrophic fractures in racing Thoroughbreds at the Hong Kong Jockey Club	cohort retrospective	Thoroughbred racehorses that raced at the HKJC over seven seasons	no	1,4,5
149	Takahashi, T. et al	2004	Japan	Association between race history and risk of superficial digital flexor tendon injury in Thoroughbred racehorses	case control	Thoroughbred racehorses that were registered with the JRA	yes	
150	Tanner, J. C. et al	2013	NZ	The association of 2-year-old training milestones with career length and racing success in a sample of Thoroughbred horses in New Zealand	cohort retrospective	NZ Thoroughbred foals	no	3,4,5
151	Thomson, P. C. et al	2014	Australia	Number, causes and destinations of horses leaving the Australian Thoroughbred and Standardbred racing industries	cross-sectional	Australian Thoroughbred and Standardbred trainers	no	3,4,5
152	Thorpe, C. T. et al	2010	UK	A review of tendon injury: Why is the equine superficial digital flexor tendon most at risk?	narrative review	All performance horses	no	1,4,5
153	Tozaki, T. et al	2019	Japan	Heritability estimates of fractures in Japanese Thoroughbred racehorses using a non-linear model	cohort retrospective	Japanese Thoroughbred racehorses	no	4,5
154	Tsuzuki, N. et al	2019	Japan	Markers for oxidative stress in the synovial fluid of Thoroughbred horses with carpal bone fracture	case control	Horses with unilateral fracture of the carpal joint bone	no	4,5
155	Turley, S. M. et al	2014	NZ	Microstructural changes in cartilage and bone related to repetitive overloading in an equine athlete model	cross-sectional	Thoroughbred racehorses	no	1,3,4,5
156	Turlo, A. J. et al	2015	Poland	The effect of different types of musculoskeletal injuries on blood concentration of serum amyloid a in thoroughbred racehorses	case control	Polish Thoroughbred racehorses	no	4,5
157	Turlo, A. J. et al	2019	Poland	Revisiting predictive biomarkers of musculoskeletal injury in thoroughbred racehorses: Longitudinal study in polish population	cohort prospective	2-year-old Polish Thoroughbred racehorses in their first training season	no	4,5
158	Turlo, A. J. et al	2018	Poland	Detecting biomarkers of musculoskeletal injury in racing thoroughbreds with nuclear magnetic resonance metabolomics	cohort retrospective	Polish Thoroughbred racehorses	no	1,4,5
159	Vallance, S. A. et al	2012	USA	Characteristics of Thoroughbred and Quarter Horse racehorses that sustained a complete scapular fracture	case control	TB racehorses that had a scapular fracture in California between 1990 and 2008	no	5
160	Vallance, S. A. et al	2013	USA	Case-control study of high-speed exercise history of Thoroughbred and Quarter Horse racehorses that died related to a complete scapular fracture	case control	TB racehorses that died between 1 January 1990 and 31 December 2008 related to a catastrophic scapular fracture	yes	
161	Varcoe-Cocks, K. et al	2006	Australia	Pressure algometry to quantify muscle pain in racehorses with suspected sacroiliac dysfunction	cross-sectional	TB racehorses	no	4,5
162	Verheyen, K. L.P. et al	2006	UK	Exercise distance and speed affect the risk of fracture in racehorses	nested case control	Racehorses throughout England	yes	
163	Verheyen, K. L. P.	2013	UK	Reducing injuries in racehorses: Mission impossible?	editorial	TB racehorses	no	1,3,4,5
164	Verheyen, K. L. P. et al	2005	UK	Training-related factors associated with dorsometacarpal disease in young Thoroughbred racehorses in the UK	cohort prospective	Racehorses throughout England	yes	
165	Verheyen, K. L. P. et al	2006	UK	A case-control study of factors associated with pelvic and tibial stress fractures in Thoroughbred racehorses in training in the UK	nested case control	Racehorses throughout England	yes	
166	Verheyen, K. L. P. et al	2003	UK	Epidemiology of fractures in British racehorses in training	cohort prospective	Racehorses throughout England	no	1,4,5
167	Verheyen, K. L. P. et al	2007	UK	Fracture rate in Thoroughbred racehorses is affected by dam age and parity	cohort prospective	Racehorses throughout England	no	4,5
168	Verheyen, K. L. P. and Wood, J. L. N.	2004	UK	Descriptive epidemiology of fractures occurring in British Thoroughbred racehorses in training	cohort prospective	Racehorses throughout England	no	4,5
169	Walsh, R. et al	2018	UK	Frequency distribution of osteochondral fragmentation of the dorsoproximal articular surface of the proximal phalanx in racing Thoroughbreds in the UK	case series	Racehorses undergoing arthroscopic surgery of the proximal phalanx	no	1,4,5
170	Watkins, K. L.	1985	UK	Epidemiology of racehorse wastage	editorial	Thoroughbred racehorses	no	1,3,4,5
171	Welsh, C. E. et al	2014	UK	Estimates of genetic parameters of distal limb fracture and superficial digital flexor tendon injury in UK Thoroughbred racehorses	cohort retrospective	UK racehorses	no	2,4,5
172	Whitton, R. C. et al	2018	Australia	Subchondral bone microdamage accumulation in distal metacarpus of Thoroughbred racehorses	cross-sectional	Thoroughbred racehorses undergoing post-mortem	no	3,4,5
173	Whitton, R. C. et al	2013	Australia	Exercise-induced inhibition of remodelling is focally offset with fatigue fracture in racehorses	cohort retrospective	Thoroughbred racehorses that died or were euthanized from April 2007 to April 2009 and underwent post-mortem examination at the University of Melbourne	no	3,4,5
174	Whitton, R. C. et al	2010	Australia	Third metacarpal condylar fatigue fractures in equine athletes occur within previously modelled subchondral bone	cohort retrospective	Thoroughbred racehorses that died or were euthanized from April 2007 to April 2009 and underwent post-mortem examination at the University of Melbourne	no	3,4,5

175	Whitton, R. C. et al	2019	Australia	Associations between pre-injury racing history and tibial and humeral fractures in Australian Thoroughbred racehorses	case control	Thoroughbred racehorses diagnosed with a fracture of the humerus or tibia by scintigraphy or at postmortem between 2002 and 2016	yes	
176	Wilsher, S. et al	2006	UK	Factors associated with failure of thoroughbred horses to train and race	cohort prospective	Thoroughbred foals	no	4,5
177	Wilson, J. H. and Robinson, R. A.	1996	USA	Risk factors for equine racing injuries	narrative review	Thoroughbred racehorses	no	1,4,5
178	Wright, I. M.	2017	UK	Racecourse fracture management. Part 3: Emergency care of specific fractures	narrative review	Throughbred racehorses with fractures	no	1,3,4,5
179	Wright, I. M. and Minshall, G. J.	2018	UK	Short frontal plane fractures involving the dorsoproximal articular surface of the proximal phalanx: Description of the injury and a technique for repair	case series	Horses with frontal plane fractures of the proximal phalanx	no	1,4,5
180	Wright, I. M. and Smith, M. R. W.	2009	UK	A lateral approach to the repair of propagating fractures of the medial condyle of the third metacarpal and metatarsal bone in 18 racehorses	case series	Thoroughbred horses with propagating fractures of the medial condyle of MC3/ MT3	no	1,4,5
181	Yang, Y. J. and Cho, G. J.	2015	Korea	Analysis of the factors influencing fractures in racehorses	cohort retrospective	Korean racehorses	no	4,5

† Criteria for exclusion at full text level

1. Review articles/Editorials, Case series/Case reports, Clinical trials or Descriptive, not analytical studies or abstracts only (n=86)*
2. Reported only race day rather than training data (n=17)
3. The outcome (MSI) was reported as morphological changes to bone/soft tissue, rather than as a clinical MSI (n=49)
4. The exposure, high-speed exercise (HSE) was not reported as one of seven measures: (n=135)
   - Total career cumulative HSE distance
   - Cumulative HSE distance 30 days before MSI
   - Cumulative HSE distance 60 days before MSI
   - Average HSE distance per day
   - Average HSE distance per event
   - Average HSE distance per 30 days
   - Total number of HSE events
5. The effect size was not reported as either an odds ratio, relative risk or hazard ratio (n=143)
