# Supplementary material for: The Effect of Combined Training and Racing High-Speed Exercise History on Musculoskeletal Injuries in Thoroughbred Racehorses: A Systematic Review and Meta-Analysis of the Current Literature
Source: Animals (Basel). 2020 Nov 11;10(11):2091. doi: 10.3390/ani10112091 (PMC7696103; doi:10.3390/ani10112091)
Supplement: Supplementary file 1 [file animals-10-02091-s004.zip › animals-965278-Supplementary File 3.pdf]

**Analysis 1 Exposure = Total cumulative high speed exercise distance (5 furlongs/1km)**											
**Study id**	**Effect size**	**Outcome**	**Authors**	**Year**	**Country**	**Title**	**Study type**	**Population**	**Total number**	**Number with outcome**	**Number without outcome**
2	OR	Fatal PSB fracture	Anthenill, L. A. et al	2007	USA	Risk factors for proximal sesamoid bone fractures associated with exercise history and horseshoe characteristics in Thoroughbred racehorses	case control	Racing Thoroughbred horses, age 2 to 5 years, that were necropsied	269	121	148
52	OR	catastrophic forelimb SAF failure	Hill, A. E. et al	2004	USA	Effects of injury to the suspensory apparatus, exercise, and horseshoe characteristics on the risk of lateral condylar fracture and suspensory apparatus failure in forelimbs of Thoroughbred racehorses	cross-sectional	Bilateral forelimb specimens distal to the antebrachiocarpal joint	268	108	160
55	OR	catastrophic MSI	Hitchens, P. L. et al	2018	USA	Relationship between historical lameness, medication usage, surgery, and exercise with catastrophic musculoskeletal injury in racehorses	case control	Thoroughbred racehorses in California	137	42	95
149	OR	SDFT injury	Takahashi, T. et al	2004	Japan	Association between race history and risk of superficial digital flexor tendon injury in Thoroughbred racehorses	case control	Thoroughbred racehorses that were registered with the JRA	1466	515	951
175	OR	stress or complete fracture of the humerus	Whitton, R. C. et al	2019	Australia	Associations between pre-injury racing history and tibial and humeral fractures in Australian Thoroughbred racehorses	case control	Thoroughbred racehorses diagnosed with a fracture of the humerus or tibia by scintigraphy or at postmortem	188	47	141
**Analysis 2 Exposure = Total cumulative high speed exercise distance (5 furlongs/1km)**											
**Study id**	**Effect size**	**Outcome**	**Authors**	**Year**	**Country**	**Title**	**Study type**	**Population**	**Total number**	**Number with outcome**	**Number without outcome**
10	HR	MSI	Bolwell, C. et al	2012	NZ	Risk factors for interruptions to training occurring before the first trial start of 2-year-old Thoroughbred racehorses	cohort prospective	2-year-old racehorses	205	19	186
121	HR	carpal injury	Reed, S. R. et al	2013	UK	Exercise affects joint injury risk in young Thoroughbreds in training	cohort prospective	Thoroughbred racehorses in UK	647	77	570
164	HR	DMD	Verheyen, K. L. P. et al	2005	UK	Training-related factors associated with dorsometacarpal disease in young Thoroughbred racehorses in the UK	cohort prospective	Racehorses throughout England	335	79	256
**Analysis 3 Exposure = Cumulative high speed exercise distance 30 days before musculoskeletal injury (furlongs)**											
**Study id**	**Effect size**	**Outcome**	**Authors**	**Year**	**Country**	**Title**	**Study type**	**Population**	**Total number**	**Number with outcome**	**Number without outcome**
2	OR	Fatal PSB fracture	Anthenill, L. A. et al	2007	USA	Risk factors for proximal sesamoid bone fractures associated with exercise history and horseshoe characteristics in Thoroughbred racehorses	case control	Racing Thoroughbred horses, age 2 to 5 years, that were necropsied	269	121	148
52	OR	catastrophic forelimb SAF failure	Hill, A. E. et al	2004	USA	Effects of injury to the suspensory apparatus, exercise, and horseshoe characteristics on the risk of lateral condylar fracture and suspensory apparatus failure in forelimbs of Thoroughbred racehorses	cross-sectional	Bilateral forelimb specimens distal to the antebrachiocarpal joint	268	108	160
55	OR	catastrophic MSI	Hitchens, P. L. et al	2018	USA	Relationship between historical lameness, medication usage, surgery, and exercise with catastrophic musculoskeletal injury in racehorses	case control	Thoroughbred racehorses in California	137	42	95
108	OR	MSI that involved the lower limbs	Perkins, N. R. et al	2005	NZ	Risk factors for musculoskeletal injuries of the lower limbs in Thoroughbred racehorses in New Zealand	cohort prospective	Racehorses training and racing in New Zealand	1146	294	852
160	OR	catastrophic scapular fracture	Vallance, S. A. et al	2013	USA	Case-control study of high-speed exercise history of Thoroughbred and Quarter Horse racehorses that died related to a complete scapular fracture	case control	TB racehorses that died between 1 January 1990 and 31 December 2008 related to a catastrophic scapular fracture	140	47	93
**Studies excluded from this analysis due to categorisation of data**											
162	OR	fracture	Verheyen, K. L.P. et al	2006	UK	Exercise distance and speed affect the risk of fracture in racehorses	nested case control	Racehorses throughout England	732	136	596
165	OR	pelvic or tibial stress fractures	Verheyen, K. L.P. et al	2006	UK	A case-control study of factors associated with pelvic and tibial stress fractures in Thoroughbred racehorses in training in the UK	nested case control	Racehorses throughout England	216	39	177
**Analysis 4 Exposure = Cumulative high speed exercise distance 60 days before musculoskeletal injury (furlongs)**											
**Study id**	**Effect size**	**Outcome**	**Authors**	**Year**	**Country**	**Title**	**Study type**	**Population**	**Total number**	**Number with outcome**	**Number without outcome**

2	OR	Fatal PSB fracture	Anthenill, L. A. et al	2007	USA	Risk factors for proximal sesamoid bone fractures associated with exercise history and horseshoe characteristics in Thoroughbred racehorses	case control	Racing Thoroughbred horses, age 2 to 5 years, that were necropsied	269	121	148
52	OR	catastrophic forelimb SAF failure	Hill, A. E. et al	2004	USA	Effects of injury to the suspensory apparatus, exercise, and horseshoe characteristics on the risk of lateral condylar fracture and suspensory apparatus failure in forelimbs of Thoroughbred racehorses	cross-sectional	Bilateral forelimb specimens distal to the antebrachiocarpal joint	268	108	160
55	OR	catastrophic MSI	Hitchens, P. L. et al	2018	USA	Relationship between historical lameness, medication usage, surgery, and exercise with catastrophic musculoskeletal injury in racehorses	case control	Thoroughbred racehorses in California	137	42	95
160	OR	catastrophic scapular fracture	Vallance, S. A. et al	2013	USA	Case-control study of high-speed exercise history of Thoroughbred and Quarter Horse racehorses that died related to a complete scapular fracture	case control	TB racehorses that died between 1 January 1990 and 31 December 2008 related to a catastrophic scapular fracture	140	47	93
**Studies excluded from this analysis due to categorisation of data**											
165	OR	pelvic or tibial stress fractures	Verheyen, K. L.P. et al	2006	UK	A case-control study of factors associated with pelvic and tibial stress fractures in Thoroughbred racehorses in training in the UK	nested case control	Racehorses throughout England	216	39	177
**Analysis 5 Exposure = Average distance high speed exercise per day (furlongs)**											
**Study id**	**Effect size**	**Outcome**	**Authors**	**Year**	**Country**	**Title**	**Study type**	**Population**	**Total number**	**Number with outcome**	**Number without outcome**
52	OR	catastrophic forelimb SAF failure	Hill, A. E. et al	2004	USA	Effects of injury to the suspensory apparatus, exercise, and horseshoe characteristics on the risk of lateral condylar fracture and suspensory apparatus failure in forelimbs of Thoroughbred racehorses	cross-sectional	Bilateral forelimb specimens distal to the antebrachiocarpal joint	268	108	160
160	OR	catastrophic scapular fracture	Vallance, S. A. et al	2013	USA	Case-control study of high-speed exercise history of Thoroughbred and Quarter Horse racehorses that died related to a complete scapular fracture	case control	TB racehorses that died between 1 January 1990 and 31 December 2008 related to a catastrophic scapular fracture	132	39	93
**Studies excluded from this analysis due to categorisation of data**											
162	OR	fracture	Verheyen, K. L.P. et al	2006	UK	Exercise distance and speed affect the risk of fracture in racehorses	nested case control	Racehorses throughout England	732	136	596
**Analysis 6 Exposure = Average distance high speed exercise per day (furlongs)**											
**Study id**	**Effect size**	**Outcome**	**Authors**	**Year**	**Country**	**Title**	**Study type**	**Population**	**Total number**	**Number with outcome**	**Number without outcome**
121	HR	metacarpo/tarsophalangeal injury	Reed, S. R. et al	2013	UK	Exercise affects joint injury risk in young Thoroughbreds in training	cohort prospective	Thoroughbred racehorses in UK	647	92	555
164	HR	DMD	Verheyen, K. L. P. et al	2005	UK	Training-related factors associated with dorsometacarpal disease in young Thoroughbred racehorses in the UK	cohort prospective	Racehorses throughout England	335	79	256
**Analysis 7 Exposure = Average distance high speed exercise per event (furlongs)**											
**Study id**	**Effect size**	**Outcome**	**Authors**	**Year**	**Country**	**Title**	**Study type**	**Population**	**Total number**	**Number with outcome**	**Number without outcome**
2	OR	Fatal PSB fracture	Anthenill, L. A. et al	2007	USA	Risk factors for proximal sesamoid bone fractures associated with exercise history and horseshoe characteristics in Thoroughbred racehorses	case control	Racing Thoroughbred horses, age 2 to 5 years, that were necropsied	269	121	148
20	OR	MSI	Cogger, N. et al	2006	Australia	Risk factors for musculoskeletal injuries in 2-year-old Thoroughbred racehorses	cohort prospective	Two-year-old Thoroughbred horses trained by participating trainers at 5 racetracks in NSW	274	110	164
55	OR	catastrophic MSI	Hitchens, P. L. et al	2018	USA	Relationship between historical lameness, medication usage, surgery, and exercise with catastrophic musculoskeletal injury in racehorses	case control	Thoroughbred racehorses in California	137	42	95
149	OR	SDFT injury	Takahashi, T. et al	2004	Japan	Association between race history and risk of superficial digital flexor tendon injury in Thoroughbred racehorses	case control	Thoroughbred racehorses that were registered with the JRA	1466	515	951
160	OR	catastrophic scapular fracture	Vallance, S. A. et al	2013	USA	Case-control study of high-speed exercise history of Thoroughbred and Quarter Horse racehorses that died related to a complete scapular fracture	case control	TB racehorses that died between 1 January 1990 and 31 December 2008 related to a catastrophic scapular fracture	132	39	93
**Analysis 8 Exposure = Average distance high speed exercise per 30 days (furlongs)**											

Study id	Effect size	Outcome	Authors	Year	Country	Title	Study type	Population	Total number	Number with outcome	Number without outcome
2	OR	Fatal PSB fracture	Anthenill, L. A. et al	2007	USA	Risk factors for proximal sesamoid bone fractures associated with exercise history and horseshoe characteristics in Thoroughbred racehorses	case control	Racing Thoroughbred horses, age 2 to 5 years, that were necropsied	269	121	148
55	OR	catastrophic MSI	Hitchens, P. L. et al	2018	USA	Relationship between historical lameness, medication usage, surgery, and exercise with catastrophic musculoskeletal injury in racehorses	case control	Thoroughbred racehorses in California	137	42	95
**Studies excluded from this analysis due to categorisation of data**											
162	OR	fracture	Verheyen, K. L.P. et al	2006	UK	Exercise distance and speed affect the risk of fracture in racehorses	nested case control	Racehorses throughout England	732	136	596
**Analysis 9 Exposure = Average distance high speed exercise per 30 days (furlongs)**											
**Study id**	**Effect size**	**Outcome**	**Authors**	**Year**	**Country**	**Title**	**Study type**	**Population**	**Total number**	**Number with outcome**	**Number without outcome**
121	HR	metacarpo/tarsophalangeal injury	Reed, S. R. et al	2013	UK	Exercise affects joint injury risk in young Thoroughbreds in training	cohort prospective	Thoroughbred racehorses in UK	647	92	555
164	HR	DMD	Verheyen, K. L. P. et al	2005	UK	Training-related factors associated with dorsometacarpal disease in young Thoroughbred racehorses in the UK	cohort prospective	Racehorses throughout England	335	79	256
**Analysis 10 Exposure = Total number of career events**											
**Study id**	**Effect size**	**Outcome**	**Authors**	**Year**	**Country**	**Title**	**Study type**	**Population**	**Total number**	**Number with outcome**	**Number without outcome**
2	OR	Fatal PSB fracture	Anthenill, L. A. et al	2007	USA	Risk factors for proximal sesamoid bone fractures associated with exercise history and horseshoe characteristics in Thoroughbred racehorses	case control	Racing Thoroughbred horses, age 2 to 5 years, that were necropsied	269	121	148
55	OR	catastrophic MSI	Hitchens, P. L. et al	2018	USA	Relationship between historical lameness, medication usage, surgery, and exercise with catastrophic musculoskeletal injury in racehorses	case control	Thoroughbred racehorses in California	137	42	95
149	OR	SDFT injury	Takahashi, T. et al	2004	Japan	Association between race history and risk of superficial digital flexor tendon injury in Thoroughbred racehorses	case control	Thoroughbred racehorses that were registered with the JRA	1466	515	951
175	OR	stress or complete fracture of the humerus	Whitton, R. C. et al	2019	Australia	Associations between pre-injury racing history and tibial and humeral fractures in Australian Thoroughbred racehorses	case control	Thoroughbred racehorses diagnosed with a fracture of the humerus or tibia by scintigraphy or at postmortem between 2002 and 2016	188	47	141
**Studies excluded from this analysis due to categorisation of data**											
160	OR	catastrophic scapular fracture	Vallance, S. A. et al	2013	USA	Case-control study of high-speed exercise history of Thoroughbred and Quarter Horse racehorses that died related to a complete scapular fracture	case control	TB racehorses that died between 1 January 1990 and 31 December 2008 related to a catastrophic scapular fracture	132	39	93
